# Reductions in Cardiovascular, Cerebrovascular, and Respiratory Mortality following the National Irish Smoking Ban: Interrupted Time-Series Analysis

**DOI:** 10.1371/journal.pone.0062063

**Published:** 2013-04-24

**Authors:** Sericea Stallings-Smith, Ariana Zeka, Pat Goodman, Zubair Kabir, Luke Clancy

**Affiliations:** 1 Institute for the Environment, Brunel University, London, United Kingdom; 2 Environmental Health Sciences Institute, Dublin Institute of Technology, Dublin, Ireland; 3 TobaccoFree Research Institute Ireland, Dublin, Ireland; 4 Department of Epidemiology and Public Health, University College Cork, Cork, Ireland; University of Chieti, Italy

## Abstract

**Background:**

Previous studies have shown decreases in cardiovascular mortality following the implementation of comprehensive smoking bans. It is not known whether cerebrovascular or respiratory mortality decreases post-ban. On March 29, 2004, the Republic of Ireland became the first country in the world to implement a national workplace smoking ban. The aim of this study was to assess the effect of this policy on all-cause and cause-specific, non-trauma mortality.

**Methods:**

A time-series epidemiologic assessment was conducted, utilizing Poisson regression to examine weekly age and gender-standardized rates for 215,878 non-trauma deaths in the Irish population, ages ≥35 years. The study period was from January 1, 2000, to December 31, 2007, with a post-ban follow-up of 3.75 years. All models were adjusted for time trend, season, influenza, and smoking prevalence.

**Results:**

Following ban implementation, an immediate 13% decrease in all-cause mortality (RR: 0.87; 95% CI: 0.76–0.99), a 26% reduction in ischemic heart disease (IHD) (RR: 0.74; 95% CI: 0.63–0.88), a 32% reduction in stroke (RR: 0.68; 95% CI: 0.54–0.85), and a 38% reduction in chronic obstructive pulmonary disease (COPD) (RR: 0.62; 95% CI: 0.46–0.83) mortality was observed. Post-ban reductions in IHD, stroke, and COPD mortalities were seen in ages ≥65 years, but not in ages 35–64 years. COPD mortality reductions were found only in females (RR: 0.47; 95% CI: 0.32–0.70). Post-ban annual trend reductions were not detected for any smoking-related causes of death. Unadjusted estimates indicate that 3,726 (95% CI: 2,305–4,629) smoking-related deaths were likely prevented post-ban. Mortality decreases were primarily due to reductions in passive smoking.

**Conclusions:**

The national Irish smoking ban was associated with immediate reductions in early mortality. Importantly, post-ban risk differences did not change with a longer follow-up period. This study corroborates previous evidence for cardiovascular causes, and is the first to demonstrate reductions in cerebrovascular and respiratory causes.

## Introduction

Exposure to secondhand smoke increases the risk of morbidity and premature mortality due to cardiovascular [1], cerebrovascular [2], and respiratory [1] causes. On March 29, 2004, the Republic of Ireland became the first country in the world to implement a national workplace smoking ban. The legislation was comprehensive, banning smoking in workplaces including restaurants, bars, and pubs.

Following the implementation of the Irish national smoking ban, studies conducted in pubs and bars demonstrated reductions in particulate concentrations [3], reductions in worker-reported exposure to secondhand smoke [4], [5], and related improvements in worker pulmonary function [3] and self-reported respiratory symptoms [5]. More recent Irish studies have shown post-ban reductions in hospital admissions due to acute coronary syndromes [6], [7] and acute pulmonary disease [7]. Epidemiological studies of the effects of comprehensive smoking bans in other countries have demonstrated reductions in mortality due to cardiovascular causes [8]–[11] and hospital admissions due to cardiovascular [11]–[20], cerebrovascular [12], [20], and respiratory causes [12], [20], [21]. Most of the studies analyzed a post-ban follow-up period of 2.5 years or less [8]–[20], with only one study analyzing a post-ban time period of 3.5 years [21]. None of the studies analyzed post-ban mortality effects in cerebrovascular or respiratory causes. The aim of this study was to assess the effect of the national smoking ban on all-cause and cause-specific, non-trauma mortality in the Republic of Ireland for the years 2000–2007.

## Methods

### Data for the Republic of Ireland

National mortality data from January 1, 2000, to December 31, 2007, were obtained from the Central Statistics Office (CSO) Ireland. From 2000–2006, mortality data were coded according to the *International Classification of Diseases, 9^th^ Revision* (ICD-9); ICD-10 codes were implemented in 2007. Primary causes of death selected for analyses included all-cause, non-trauma mortality (ICD-9 codes 001-799/ICD-10 codes A00-R99), smoking-related mortality including all cardiovascular diseases (390-429/I01-I52), ischemic heart disease (IHD) (410-414, 429.2/I20-I25), acute myocardial infarction (AMI) (410/I21), stroke (430-438/I60-I69), all respiratory diseases (460-519/J0-J99), and chronic obstructive pulmonary disease (COPD) (490–492, 494–496/J40–J44, J47). Non-smoking related mortality (001–389, 440–459, 520–799/A00–H95, I26–I52, K00–R99) was included as a control.

Age and gender-specific population estimates for the census years 2002 and 2006 were obtained from the CSO Ireland [22].

### Statistical Analyses

Poisson regression with interrupted time-series analysis was used to calculate weekly mortality rates. The average of age and gender-specific population figures for census years 2002 and 2006 was included as an offset in the models. Time was defined as a continuous variable from week 1 of 2000 to week 51 of 2007 and was included in the model to capture long-term trends in mortality rates over time. Week 0 of 2000 and week 52 of 2007 were excluded from analyses as some days fell in other calendar years.

An indicator variable was used to define the smoking ban, with a value of zero given to the weeks before ban implementation and a value of one given to the week of ban implementation (beginning March 28, 2004) and all following weeks. An interaction term between the smoking ban and time was defined to estimate the monotonic change in the post-ban period. The analysis was restricted to mortality events in age groups ≥35 years to reflect the population at risk for smoking-related mortality.

Significant Durbin-Watson statistics indicated negative first-order autocorrelation for the mortality data. To account for this, terms specifying a first-order autoregressive structure were applied to all models.

In 2007, the change in coding scheme from ICD-9 to ICD-10 resulted in a 43% decrease in pneumonia/influenza mortality compared to 2006. Since roughly 49% of all respiratory mortality was comprised of pneumonia/influenza over the 2000-2006 study period, the large decrease in 2007 affected data reliability for this category. Therefore, 2007 data were excluded from analyses of all respiratory mortality. No other causes of death were affected by the coding change.

To detect any differences between short-term and long-term post-ban mortality effects, an additional indicator variable was included in final models. Values of one were given for the week of ban implementation and the subsequent weeks up to one, three, six, or twelve months post-ban, with all other weeks denoted by a value of zero.

For further validation that the final models were detecting true ban effects, three additional models were refitted with false smoking ban implementation dates set at six months, one year, and two years pre-ban.

A peak in observed mortality was detected during the winter of 1999–2000; therefore, two additional models were tested to determine whether the full inclusion (beginning December 1999) or full exclusion (beginning April 2000) of the winter season influenced model results.

To determine the modifying effects of age and gender on the smoking ban-mortality association, analyses were stratified for ages 35–64 years, ages 65–84 years, and ages ≥85 years, males, and females. Due to the small number of events in each subcategory, it was not possible to stratify by age and gender simultaneously.

### Potential Confounders

Adjustments were made for temporal changes in season [23], [24], influenza activity [25], and national smoking prevalence. Season was designated based upon calendar weeks with winter defined as December-February, spring as March-May, summer as June-August, and autumn as September-November. Seasonal adjustment with annual and semi-annual sine and cosine terms yielded similar effects.

Weekly surveillance data for influenza-like illnesses (ILI) were available from the Irish Health Protection Surveillance Centre [26] for the influenza seasons (October-May) of 2000–2001 to 2007–2008. ILI activity for the influenza season of 1999–2000 was approximated using published data from the European Influenza Surveillance Scheme [25]. Periods of high ILI activity were defined as weeks when the reported rate of ILI was ≥60/100,000, roughly twice the background rate of ILIs for the Republic of Ireland.

Monthly smoking prevalence data from a nationally representative computer-assisted telephone survey of 1,000 persons per month, ages ≥15 years, were obtained from the Ireland Office of Tobacco Control (OTC) [27]. Data were available for the months of July 2002–December 2007. A linear regression fitted to OTC data was used to approximate smoking prevalence for 2000–2001. Annual averages were calculated to adjust for smoking prevalence in all models.

### Presentation of Results

The Poisson model equation estimating weekly morality rates was expressed as follows: 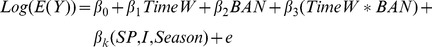
(1)where *Y* denotes the response (weekly mortality), *β_0_* is the model intercept, *β_1_* is the model coefficient for the weekly time trend variable, *β_2_* is the coefficient of the indicator variable for smoking ban policy implementation, *β_3_* is the coefficient of the interaction between the indicator variable for *BAN* and the weekly time trend, *β_k_* denotes the effects for a set of covariates of interest (*smoking prevalence-SP, influenza-I, and Season*), and *e* is the model error term.

**Figure 1:**
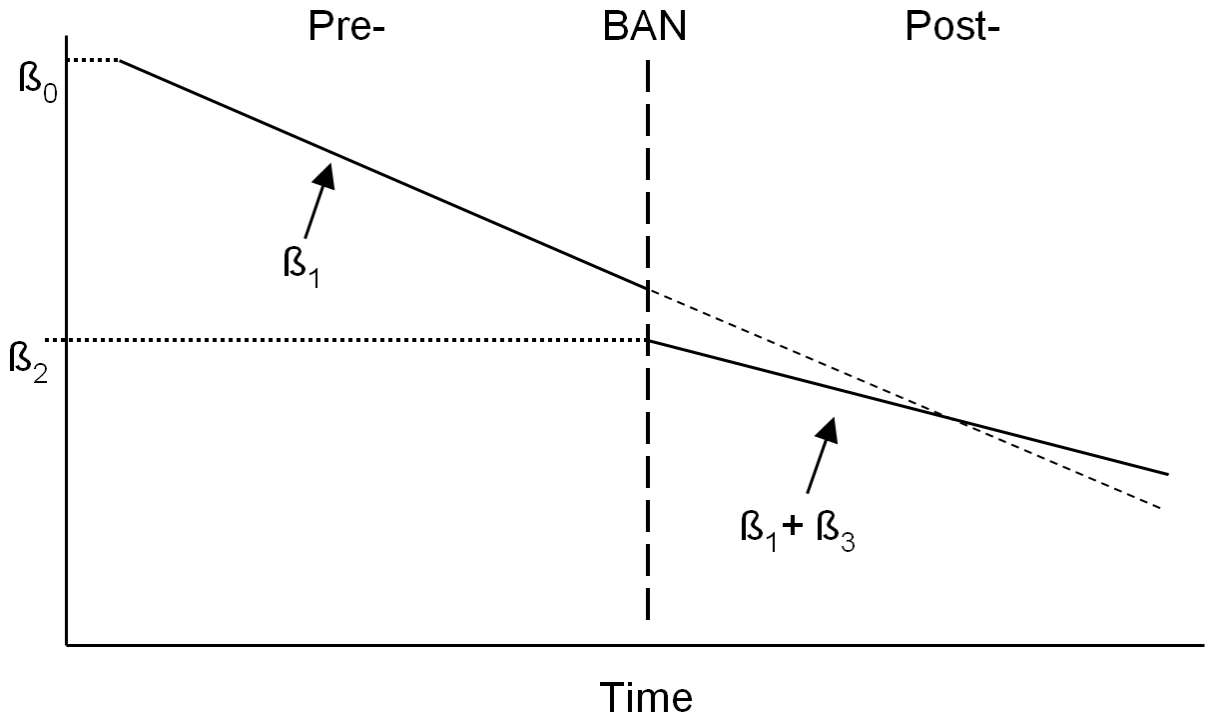
Interpretation of model results. The Y axis represents weekly mortality, the X axis represents Time, and the bold line represents the monotonic mortality change pre- and post-ban. The vertical interrupted line represents the time of the smoking ban policy implementation.



In the pre-ban period (Figure 1), *Ban*  = 0, and the model takes the form:

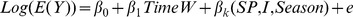
(2)In the post-ban period, *Ban*  = 1, thus the model takes the form: 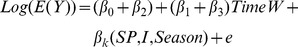
(3)where *β_2_* is the change in the log rate ratio for the immediate effect of the smoking ban and (*β_1_* + *β_3_*) is the post-ban rate of change in mortality, with *β_3_* representing the change in slope after the ban. For results presentation, rate ratios (RR) were calculated for the immediate effect coefficients as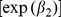, and weekly trend effect coefficients were converted to annual change with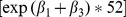. The 95% confidence intervals (CI) for the annual trend effect accounted for the overall variance of the pre- and post-ban slopes with the formula 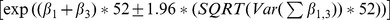, with 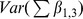 determined as 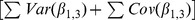, where 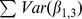 is the sum of pre- and post-ban slope variance and 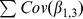 is the sum of the pre- and post-ban slope covariance [28], [29].

All analyses were conducted using SAS version 9.2, and statistical modeling was carried out with the SAS GLIMMIX procedure, allowing adjustment for autocorrelation [30].

### Number of Deaths Prevented

The predicted incremental number of deaths that would have occurred in the absence of a national smoking ban for each of the 3.75 post-ban years (April 2004–December 2007) were calculated as follows: Predicted deaths*_i_*  =  [Observed deaths*_j_* - (Observed deaths_j_*Pre-ban annual change)], where *i* represents each post-ban year, and *j* denotes the number of annual deaths from the preceding year.

### Active Smoking Attributable Risk

To determine the extent to which observed mortality reductions in the first post-ban year were attributable to decreases in active smoking, the appropriate relative risks for IHD, stroke, and COPD in active and former smokers were derived from the published literature [31]–[36] and applied to an attributable risk formula previously published by Barone-Adesi *et al* [31]. To support these analyses, crude and model-estimated changes in pre- and post-ban monthly smoking prevalence were calculated to determine whether ban implementation affected smoking prevalence in the population.

## Results

**Figure 2:**
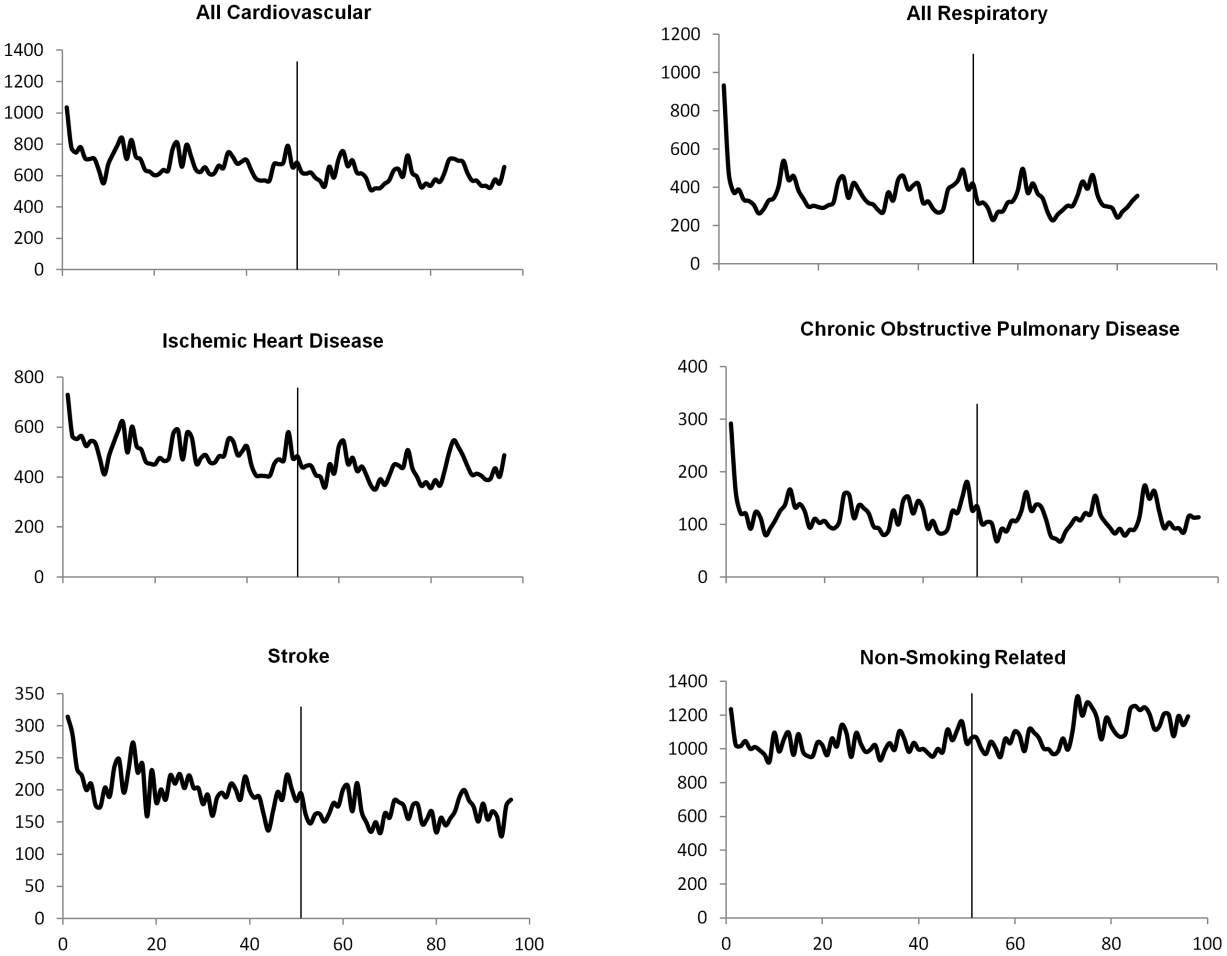
Observed Monthly Mortality in the Republic of Ireland, 2000–2007.* *All Respiratory excludes data from year 2007. The vertical line represents the month of smoking ban implementation. Monthly mortality is displayed rather than weekly for a clearer visual representation of trends.

During the study period, 215,878 non-trauma deaths occurred in the Irish population ages ≥35 years. The population at risk was 1.9 million, mean figures from the 2002 and 2006 censuses. Seasonal variations in mortality were detected, with the largest number of deaths occurring in autumn and winter. From 2000–2007, an overall decrease in mortality rates was observed for all smoking-related causes of death with decreases becoming more pronounced in the post-ban period (Figures 2 and S1). In contrast, non-smoking related mortality showed a sharp increase in 2006 which continued throughout the end of the study period.

From 2000–2007, five periods of increased ILI activity were identified, with the largest period of increase occurring for eight consecutive weeks in the latter part of the 2000–2001 influenza season.

Results of the Poisson regression analyses demonstrated that all-cause mortality rates decreased in the pre-ban period (RR: 0.98; 95% CI: 0.96–0.99). Similar pre-ban reductions were found for all cardiovascular causes, IHD, AMI, stroke, and COPD. No pre-ban mortality effects were seen in the all respiratory category or for non-smoking related mortality (data not shown).

Overall and gender-specific post-ban results of Poisson regression analyses are reported in Table 1. Following the implementation of the ban, an immediate 13% decrease in all-cause mortality was observed (RR: 0.87; 95% CI: 0.76–0.99). Likewise, an immediate 26% reduction in mortality was seen in IHD (RR: 0.74; 95% CI: 0.63–0.88), a 32% reduction in stroke (RR: 0.68; 95% CI: 0.54–0.85), and a 38% reduction in COPD (RR: 0.62; 95% CI: 0.46–0.83). IHD and stroke reductions were observed in both genders, but reductions in all respiratory mortality were seen only in females (RR: 0.64; 95% CI: 0.42–0.98) driven by reductions in COPD (RR: 0.47; 95% CI: 0.32–0.70).

**Table 1:** Multivariate Analysis† of Overall and Gender-Specific Post-Ban Effects on Mortality Rates‡, Ages ≥35 years, Republic of Ireland, 2000–2007.

	Overall		Males		Females	
Cause of Death	Immediate Effects	Gradual Effects per Annum	Immediate Effects	Gradual Effects per Annum	Immediate Effects	Gradual Effects per Annum
	RR (95% CI)	RR (95% CI)	RR (95% CI)	RR (95% CI)	RR (95% CI)	RR (95% CI)
**All-Cause Mortality**	0.87 (0.76–0.99)	1.01 (0.98–1.05)	0.87 (0.76–1.00)	1.01 (0.98–1.05)	0.86 (0.74–0.99)	1.01 (0.98–1.05)
**All Cardiovascular**	0.86 (0.74–1.00)	0.99 (0.95–1.03)	0.85 (0.72–1.02)	0.99 (0.95–1.03)	0.87 (0.72–1.04)	0.99 (0.95–1.04)
**Ischemic Heart Disease**	0.74 (0.63–0.88)	1.00 (0.96–1.04)	0.71 (0.58–0.86)	1.01 (0.96–1.05)	0.79 (0.64–0.97)	1.00 (0.95–1.05)
**Acute Myocardial Infarction**	0.89 (0.74–1.08)	0.97 (0.92–1.02)	0.87 (0.70–1.10)	0.97 (0.91–1.02)	0.92 (0.71–1.18)	0.97 (0.91–1.03)
**Stroke**	0.68 (0.54–0.85)	1.00 (0.95–1.05)	0.66 (0.49–0.89)	0.99 (0.92–1.06)	0.69 (0.53–0.91)	1.01 (0.94–1.07)
**All Respiratory** *	0.77 (0.54–1.10)	1.01 (0.92–1.10)	0.93 (0.63–1.38)	0.98 (0.89–1.07)	0.64 (0.42–0.98)	1.04 (0.94–1.14)
**COPD**	0.62 (0.46–0.83)	1.03 (0.96–1.11)	0.78 (0.55–1.12)	1.01 (0.92–1.09)	0.47 (0.32–0.70)	1.07 (0.97–1.16)
**Non-Smoking Related Mortality**	0.85 (0.75–0.97)	1.05 (1.02–1.08)	0.85 (0.73–1.00)	1.06 (1.02–1.09)	0.84 (0.71–0.99)	1.04 (1.00–1.08)

† Adjusted for time trend, season, influenza, and smoking prevalence.

‡ Age and gender-standardized according to average census population figures for 2002 and 2006.

* All Respiratory excludes data from year 2007.

In contrast, an immediate 15% decrease was observed for non-smoking related mortality (RR: 0.85; 95% CI: 0.75–0.97), followed by a 5% increase each post-ban year (RR: 1.05; 95% CI: 1.02–1.08). This resulted in a net post-ban increase of 4%. No annual trend effects in post-ban mortality were detected for any smoking-related causes of death.

Table 2 displays Poisson regression results stratified by age category. For ages 35–64 years, an immediate post-ban decrease in all-cause mortality was observed (RR: 0.79; 95% CI: 0.67–0.93), followed by annual trend increases in all-cause mortality (RR: 1.06; 95%: 1.02–1.10), resulting in a net post-ban increase of 2%. For ages 65–84 years, immediate decreases were seen in all-cause mortality (RR: 0.87; 95% CI: 0.75–0.99). Similar immediate decreases were observed in IHD, stroke, and COPD for ages 65–84 years and for ages ≥85 years.

**Table 2:** Multivariate Analysis† of Post-Ban Effects on Mortality Rates by Age Category‡, Republic of Ireland, 2000–2007.

	Ages 35–64 Years		Ages 65–84 Years		Ages ≥85 Years	
Cause of Death	Immediate Effects	Gradual Effects per Annum	Immediate Effects	Gradual Effects per Annum	Immediate Effects	Gradual Effects per Annum
	RR (95% CI)	RR (95% CI)	RR (95% CI)	RR (95% CI)	RR (95% CI)	RR (95% CI)
**All-Cause Mortality**	0.79 (0.67–0.93)	1.06 (1.02–1.10)	0.87 (0.75–0.99)	0.99 (0.96–1.03)	0.94 (0.80–1.10)	1.02 (0.98–1.06)
**All Cardiovascular**	0.91 (0.69–1.20)	0.98 (0.91–1.05)	0.86 (0.73–1.02)	0.97 (0.93–1.01)	0.86 (0.70–1.05)	1.03 (0.98–1.08)
**Ischemic Heart Disease**	0.74 (0.53–1.02)	1.01 (0.93–1.08)	0.74 (0.61–0.89)	0.98 (0.94–1.03)	0.78 (0.62–0.99)	1.04 (0.99–1.10)
**Acute Myocardial Infarction**	0.89 (0.58–1.36)	0.97 (0.86–1.07)	0.90 (0.71–1.13)	0.94 (0.89–1.00)	0.94 (0.71–1.26)	1.01 (0.94–1.09)
**Stroke**	0.66 (0.37–1.18)	1.00 (0.86–1.14)	0.75 (0.57–0.99)	0.96 (0.90–1.03)	0.61 (0.44–0.83)	1.05 (0.97–1.13)
**All Respiratory** *	1.59 (0.75–3.39)	0.93 (0.75–1.11)	0.71 (0.47–1.05)	1.01 (0.91–1.10)	0.75 (0.48–1.17)	1.03 (0.92–1.14)
**COPD**	0.74 (0.32–1.72)	1.04 (0.83–1.24)	0.68 (0.48–0.96)	1.01 (0.92–1.09)	0.49 (0.31–0.78)	1.09 (0.98–1.20)
**Non-Smoking Related Mortality**	0.72 (0.60–0.86)	1.10 (1.06–1.14)	0.86 (0.75–1.00)	1.03 (0.99–1.06)	1.03 (0.85–1.25)	1.05 (1.01–1.10)

† Adjusted for time trend, season, influenza, and smoking prevalence.

‡ Age and gender-standardized according to average census population figures for 2002 and 2006.

* All Respiratory excludes data from year 2007.

The inclusion of additional post-ban indicator variables at one, three, six, and twelve months implied only short-term ban effects. The testing of false ban implementation dates showed that immediate mortality effects were either non-significant or smaller in magnitude compared to actual ban effects. Only AMI showed a larger effect with the false date of one year pre-ban which coincided with the announcement by the Irish Minister for Health that a ban was to come into force on March 29, 2004. Additional sensitivity analyses demonstrated that model results were largely unaffected by the 1999–2000 winter peak in mortality, with no differences in effects observed for any smoking-related causes of death.

In the absence of a national smoking ban, an estimated 3,726 (95% CI: 2,305–4,629) additional smoking-related deaths would have occurred. This crude estimate indicates that reductions occurred in respiratory (up to 2006, 1,896 deaths; 95% CI: 1,517–2,152), cardiovascular (1,508 deaths; 95% CI: 690–1,926), and stroke mortality (322; 95% CI: 98–552). No deaths were prevented in association with non-smoking related mortality.

The resulting mortality decreases following smoking ban implementation were primarily due to reductions in passive smoking. The attributable risk calculations for IHD, stroke, and COPD respectively demonstrated that <1% of smoking ban effects was due to decreases in active smoking. Additional analyses assessing the change in smoking prevalence in the Irish population as a result of ban implementation showed no observable effects.

## Discussion

The implementation of a national comprehensive smoking ban in the Republic of Ireland was associated with immediate mortality reductions in the population aged ≥35 years. After adjusting for time trend, seasonal variation, periods of high influenza activity, and national smoking prevalence, immediate post-ban reductions were observed in all-cause, IHD, stroke, and COPD mortality, indicating that the immediate removal of exposure to passive smoking was effective in preventing early mortality in the population most at risk.

No gradual post-ban trend effects were seen in any smoking-related causes of death. These findings are compatible with a similarly-designed analysis of the effects of the Scottish national smoking ban on pregnancy complications, which detected immediate step change reductions, but no gradual effects [37]. Importantly, the most recent meta-analysis of 45 smoking ban effect studies demonstrated that post-ban risk differences in cardiovascular deaths and hospital admissions for cardiovascular, cerebrovascular, and respiratory diseases did not change with a longer follow-up period [38]. This provides strong epidemiological evidence that smoking ban effects are seen immediately rather than gradually.

The decreases in IHD and stroke mortality were evident in both genders, while decreases in all respiratory and COPD mortality were noted for females. Stratification by age categories demonstrated cause-specific reductions for IHD, stroke, and COPD in ages ≥65 years. In contrast, for non-smoking related mortality both immediate post-ban reductions and gradual trend effects were detected, resulting in an overall post-ban increase.

The post-ban IHD reductions are consistent with the findings of two Irish studies, one of which demonstrated 12% reductions in hospital admissions due to acute coronary syndrome (ACS) in the first year following smoking ban implementation, with further reductions of 13% in the third post-ban year [6] and the other which showed an 18% decrease in ACS admissions for the oldest age groups (50–69 years) in the two post-ban years compared to the two pre-ban years [7]. Additional corroborative evidence from other countries includes decreases in out-of-hospital deaths due to acute coronary events for ages 35–64 years (15%) and ages 65–74 years (16%) one year following implementation of the Italian national smoking ban [10] and a 6% decrease in out-of-hospital ACS deaths in the 10 months following implementation of the Scottish national smoking ban [11].

This study has been the first to demonstrate post-ban reductions in stroke mortality. These findings are corroborated by prior studies reporting post-ban reductions in stroke hospital admissions. One study assessing the effects of a three-phase, province-wide smoking ban in Ontario, Canada, reported a 24% reduction in stroke hospital admissions following the second phase of the legislation, which expanded the existing partial workplace ban to include restaurants [20]. A study evaluating the effects of the Arizona statewide smoking ban demonstrated decreases in hospital admissions due to acute stroke (14%; *p* = 0.001) in the first post-ban year for counties that did not have prior local smoking legislation in place [12]. Although a study of the effects of the New York statewide smoking ban found no effects in stroke admissions in the first post-ban year, the authors suggested that previously enforced local smoking restrictions resulted in low secondhand smoke exposure among residents [18].

This has also been the first study to report smoking ban effects on respiratory mortality, with decreases in all respiratory mortality detected in females and decreases in COPD mortality detected overall, in females, and in persons aged ≥65 years. These findings are supported by a study that reported overall decreases in COPD hospital admissions following a province-wide smoking ban in Ontario, Canada [20]. Although a recent Irish study did not find post-ban decreases in COPD hospital admissions in the two post-ban years compared to the two pre-ban years, a 15% decrease in overall pulmonary admissions was detected in the same period [7].

The public health importance of the Irish national smoking ban is strongly demonstrated in estimates of the number of deaths prevented in the post-ban years. There were 3,726 fewer smoking-related deaths than would have been expected in the absence of a smoking ban. This number of prevented deaths is slightly attenuated when compared to the immediate percent reductions represented by the model, considering that model estimates account for gradual trends and other contributing factors.

The results of the attributable risk calculations for IHD mortality, which demonstrated that <1% of smoking ban effects was due to decreases in active smoking, are in concurrence with the findings of two studies that assessed the cardiovascular health effects of the national Italian smoking ban [10], [31]. These studies showed that 0.7% of the estimated post-ban reductions in hospital admissions due to AMI [31] and <2% of the estimated post-ban reductions in hospital admissions and out-of-hospital deaths due to acute coronary events [10] were due to changes in active smoking in the first post-ban year.

Attributable risk calculations also showed that reductions in active smoking accounted for <1% of post-ban reductions in stroke and COPD mortality, but no corroborative studies are available to make comparisons as this is the first study to assess post-ban effects in these mortality outcomes. Nevertheless, these results were supported in that no observable change in smoking prevalence was seen in Ireland as a result of the ban. Together, these findings suggest that mortality benefits were the result of reductions in exposure to passive smoking.

Rapid physiological changes occur within minutes to hours of exposure to passive smoke, increasing risk of adverse cardiovascular and cardiopulmonary events [39], [40] and resulting in effects in non-smokers that are 80% to 90% as large as those experienced by chronic, active smokers [41]. Exposure to even low levels of passive smoke decreases oxygen delivery to the heart as the carbon monoxide from cigarette smoke competes with oxygen for binding sites on red blood cells [42]. This impaired oxygen delivery to the heart particularly affects persons with existing cardiovascular disease, increasing arrhythmias and causing ischemia [42]. This and other related evidence has resulted in the recommendation that clinicians advise the families of patients with existing cardiovascular disease not to smoke while the patient is present [35], [39]. The implementation of the national Irish smoking ban resulted in an immediate removal of exposure to passive smoke in workplaces and public places, therefore likely reducing population risk of experiencing the aforementioned triggers of an adverse cardiovascular or cardiopulmonary event, particularly for those with existing disease.

Information from the Survey of Lifestyle, Attitudes, and Nutrition (SLÁN), a national survey of the Irish population ages ≥18 years, was used to investigate trends in cardiovascular and respiratory risk factors over the study period. For the years 1998 and 2002, SLÁN data were collected through self-administered, postal questionnaires, but in 2007, data were collected through face-to-face interviews conducted in the homes of respondents. As such, 2007 figures may not be directly comparable to those of previous survey waves. However, obesity prevalence and levels of physical activity remained steady across the study period. The percentage of persons consuming over the recommended weekly alcohol limit decreased in the 2007 survey wave; however, this result should be interpreted with caution as persons may have been less likely to report high levels of alcohol consumption in face-to-face interviews [43].

Due to excise tax increases in Ireland, cigarettes prices increased by more than 10% in 2000, 2003, and 2007; however, the estimated effects on smokers aged ≥35 years were minimal [44]. In 2002, Ireland adopted non-graphic, non-pictorial, health warnings for cigarette packages, and extended the existing TV and print media advertising ban to include selected types of indirect advertising [44]. The advertising ban was further extended in 2004, but product placements and certain forms of sponsorship were still allowed [44]. Although these additional tobacco control interventions may have resulted in synergistic health improvements with the national smoking ban, their estimated effects were small and gradual and are thus insufficient to explain the large mortality reductions detected immediately following the implementation of the national smoking ban.

A few limitations of this study should be addressed. Direct adjustments for weather were not possible due to the small number of weekly mortality events remaining after stratification by age, gender, and region. Nonetheless, adjustment for seasonal variation in all models partially accounts for weather effects. Likewise, data limitations prevented assignment of air pollution measures to individual mortality events. However, following implementation of a series of coal bans across Ireland's major cities from 1990–2000, large declines in black smoke were noted [45], along with subsequent reductions in cardiovascular and respiratory mortality in Dublin [46]. These air quality improvements may partly explain the pre-ban mortality decreases detected in this study.

A key strength of this study was the use of time-series analysis, which accounts for secular trends by design and is therefore the strongest method for assessing the effects of a broad-based intervention such as a national policy change [47]. Additionally, this study was unique in that post-ban effects in multiple causes of death were examined, including deaths due to cerebrovascular and respiratory diseases which have not been reported in any prior studies, and the post-ban follow-up period was more extensive than any other previously reported.

## Conclusion

The national smoking ban in the Republic of Ireland was associated with immediate reductions in early mortality, with specific benefits observed in cardiovascular, cerebrovascular, and respiratory causes. Importantly, post-ban risk differences did not change with a longer follow-up period. As a result of the ban, unadjusted estimates indicate that 3,726 smoking-related deaths were likely prevented. This study provides further evidence of the large public health impacts of smoking ban legislation.

## Supporting Information

Figure S1 Weekly Age and Gender-Standardized Mortality Rates, Republic of Ireland, 2000-2007.* *All Respiratory excludes data from year 2007. The vertical line represents the week of smoking ban implementation.
(TIFF)
