# Supplementary figures and images for: Reductions in Cardiovascular, Cerebrovascular, and Respiratory Mortality following the National Irish Smoking Ban: Interrupted Time-Series Analysis
Source: PLoS One. 2013 Apr 24;8(4):e62063. doi: 10.1371/journal.pone.0062063 (PMC3634756; doi:10.1371/journal.pone.0062063)

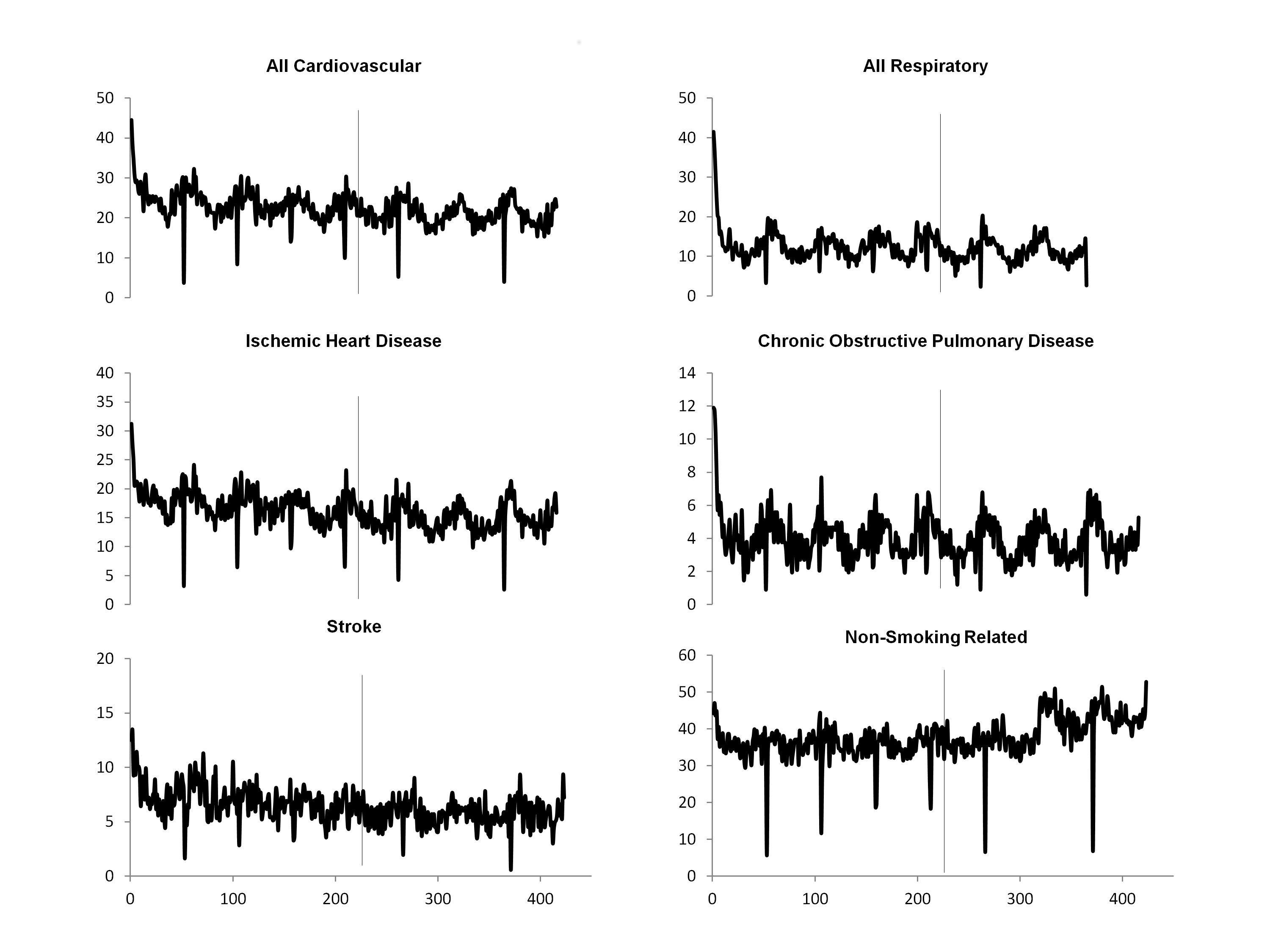

Supplement: Figure S1 — Weekly Age and Gender-Standardized Mortality Rates, Republic of Ireland, 2000-2007.* *All Respiratory excludes data from year 2007. The vertical line represents the week of smoking ban implementation. (TIFF) [file pone.0062063.s001.tiff]
